# Conserved microRNA editing in mammalian evolution, development and disease

**DOI:** 10.1186/gb-2014-15-6-r83

**Published:** 2014-06-25

**Authors:** Maria Warnefors, Angélica Liechti, Jean Halbert, Delphine Valloton, Henrik Kaessmann

**Affiliations:** 1 Center for Integrative Genomics, University of Lausanne, 1015 Lausanne, Switzerland; 2 Swiss Institute of Bioinformatics, 1015 Lausanne, Switzerland

## Abstract

**Background:**

Mammalian microRNAs (miRNAs) are sometimes subject to adenosine-to-inosine RNA editing, which can lead to dramatic changes in miRNA target specificity or expression levels. However, although a few miRNAs are known to be edited at identical positions in human and mouse, the evolution of miRNA editing has not been investigated in detail. In this study, we identify conserved miRNA editing events in a range of mammalian and non-mammalian species.

**Results:**

We demonstrate deep conservation of several site-specific miRNA editing events, including two that date back to the common ancestor of mammals and bony fishes some 450 million years ago. We also find evidence of a recent expansion of an edited miRNA family in placental mammals and show that editing of these miRNAs is associated with changes in target mRNA expression during primate development and aging. While global patterns of miRNA editing tend to be conserved across species, we observe substantial variation in editing frequencies depending on tissue, age and disease state: editing is more frequent in neural tissues compared to heart, kidney and testis; in older compared to younger individuals; and in samples from healthy tissues compared to tumors, which together suggests that miRNA editing might be associated with a reduced rate of cell proliferation.

**Conclusions:**

Our results show that site-specific miRNA editing is an evolutionarily conserved mechanism, which increases the functional diversity of mammalian miRNA transcriptomes. Furthermore, we find that although miRNA editing is rare compared to editing of long RNAs, miRNAs are greatly overrepresented among conserved editing targets.

## Background

MicroRNAs (miRNAs) are short RNAs (approximately 23 nucleotides) that downregulate gene expression by binding to target mRNAs, thereby inducing mRNA destabilization or translational repression [1]. Mammalian genomes typically give rise to several hundred distinct miRNAs [2,3], many of which are highly conserved, even between distantly related species [3,4]. In particular, there is strong purifying selection on the ‘seed’ region, which corresponds to nucleotides 2 to 7/8 of the miRNA and is the main determinant of target specificity [1,3,5]. Considering this evolutionary constraint, it is perhaps surprising that several miRNA variants, or ‘isomiRs’, are sometimes produced from the same locus, including variants with seed alterations [6].

One mechanism through which such miRNA diversity can be created is RNA editing, where individual bases within an RNA transcript are chemically modified in such a way that the RNA sequence no longer corresponds to its genomic template. In mammals, the most common form of RNA editing is catalyzed by two adenosine deaminases acting on RNA (ADARs), known as ADAR and ADARB1 (or ADAR1 and ADAR2); both enzymes target double-stranded RNA (dsRNA) and are able to convert adenosine (A) into inosine (I), a base with base-pairing properties similar to those of guanosine (G) [7]. Although mature miRNAs are single-stranded, they are excised from a longer precursor with a characteristic double-stranded stem-loop structure, which can serve as a substrate for ADAR enzymes [8-10]. ADARs do not edit indiscriminately, however; A-to-G mismatches indicative of A-to-I editing are found only for a subset of all miRNAs and, within those, only at specific sites [11]. The precise factors that influence miRNA editing specificity are only partially understood [12].

In humans, most edited sites in mature miRNAs are located within the seed [13] and editing therefore has the potential to redirect these miRNAs to new target genes. Studies of the miRNA miR-376a-1 have demonstrated that genes downregulated by the edited form are not affected by the unedited form and vice versa [11], and that the expression of target genes covaries with miRNA editing frequencies during mouse development [14]. Moreover, editing-induced rewiring of the miR-376a-1 regulatory network is of medical interest, since absence of the edited miRNA has been shown to promote invasiveness of human gliomas [15]. In addition to the effects on target specificity, miRNA editing can also affect miRNA expression levels by preventing proper processing by Drosha or Dicer during miRNA biogenesis [9,16]. A recent survey of miRNA expression in mouse embryos identified approximately one-third of all miRNAs as upregulated in embryos deficient in ADAR and ADARB1 compared to wild-type, although it is possible that these differences arise independently of miRNA editing, as might be the case if ADARs interfere with the miRNA processing pathway simply by binding to the RNA [17].

Some miRNAs are subject to A-to-I editing at identical positions in human and mouse [11-13,18], but the evolutionary conservation of miRNA editing beyond placental mammals has not been investigated. In this study, we have therefore compiled an atlas of conserved miRNA editing repertoires in six core species (human, macaque, mouse, opossum, platypus and chicken) and several complementary datasets. For our catalog of edited miRNAs, we traced their evolutionary origins and characterized to what extent the identified sites were edited in different tissues, developmental stages and disease states. We show that miRNA editing is highly dynamic within a single individual, but that broad editing patterns persist across species, suggesting that the added layer of regulatory complexity introduced by miRNA editing is an integral and evolutionarily stable feature of mammalian transcriptomes.

## Results

### Detection of shared miRNA editing events

To identify conserved miRNA editing events, we mined small RNA sequencing data from human, rhesus macaque, mouse, opossum, platypus and chicken [3] for RNA-DNA mismatches, using a set of stringent criteria (Materials and methods). Briefly, we only considered reads where the sequencing error rate was below 0.1% for all positions and that aligned to the respective genome with no more than one mismatch. We then identified sites within known miRNAs [2,3] with a tissue-specific RNA-DNA mismatch frequency above 5% and at least five reads for each variant, in at least two species. We found 10 such sites (Table 1), none of which overlapped with known human SNPs [19]. All mismatches were A-to-G, as expected for canonical A-to-I editing, indicating that our method allowed genuine editing events to be distinguished from sequencing errors. Among the identified sites, six were experimentally validated targets of ADAR enzymes in human or mouse [11-13] and an additional two had previously been identified as editing candidates using deep sequencing of mouse samples [13,18], which further confirmed that our pipeline could reliably identify *bona fide* miRNA editing events directly from small RNA sequencing data. By taking evolutionary conservation into account, it is therefore possible to investigate miRNA editing also in non-model species that lack SNP information and for which it is not feasible to perform extensive validation experiments.

**Table 1:** Detected miRNA editing events that were shared between at least two species

**ID**	**Pos.**	**Human**	**Macaque**	**Mouse**	**Opossum**	**Platypus**	**Chicken**	**Human SNP**	**Opossum SNP**	**Known**	**Validated**
miR-27a	6	>1%	>1%			>1%		No	-	Human^e^	-
										-	-
miR-99b*	2		>1%	>1%				No	-	Human^c^	-
										Mouse^c^	Mouse^c^
miR-140*	16				>5%		>5%	No	No	-	-
										-	-
miR-187*	5			>1%	>1%			No	No	-	-
										-	-
miR-301a	20		>1%		>5%		>5%	No	No	-	-
										-	-
miR-376a-1	3	>1%	>1%	>1%				No	-	Human^b,c,d^	-
										Mouse^b,c,d,e,f^	Mouse^b,c^
miR-376b	6	>5%		>5%				No	-	Human^b,c,d^	-
										Mouse^b,c,d,f,g^	Mouse^b,c^
miR-376c	6	>5%		>5%				No	-	Human^e^	-
										Mouse^b,d,e,f^	Mouse^b^
miR-379	5		>5%	>5%				No	-	Human^a,c,e^	-
										Mouse^c,d,e,f^	Mouse^c^
miR-381	4	>5%	>5%	>5%				No	-	Human^e^	Human^e^
										Mouse^d,f,g^	-
miR-411	5	>5%	>5%	>5%				No	-	Human^b,d^	-
										Mouse^c,d,e^	Mouse^c^
miR-455	17		>1%				>1%	No	-	Human^e^	Human^e^
										-	-
miR-497	2	>5%	>5%	>5%	>5%			No	No	Human^e^	Human^e^
										Mouse^d,e^	-
miR-497*	20	>5%	>5%					No	No	Mouse^d^	-
										-	-
miR-1251	6		>5%	>5%	>1%			No	No	Mouse^d,e^	-
										-	-

Summary of the output from the miRNA editing detection pipeline, run with a 5% or 1% frequency cutoff. The two leftmost columns specify the miRNA ID and the position at which editing was observed. The following six columns show the detected editing events. For estimates of editing frequencies in individual samples, please refer to Figure 3 and Table S2 in Additional file 2. SNP data for human were taken from dbSNP [19], while opossum SNPs were investigated through Sanger sequencing (see Materials and methods). The two rightmost columns summarize whether the identified sites had previously been reported (‘known’) and experimentally validated (‘validated’) in human and mouse.

^a^ Blow *et al*. [10].

^b^ Kawahara *et al*. [11].

^c^ Kawahara *et al*. [12].

^d^ Chiang *et al*. [18].

^e^ Alon *et al*. [13].

^f^ Ekdahl *et al*. [14].

^g^ Vesely *et al*. [17].

In addition to the 10 high-confidence sites, we included 5 sites with A-to-G mismatch frequencies above 1% in at least two species, which had either been identified in previous studies of miRNA editing (Table 1) or for which we could confirm the absence of genomic SNPs by Sanger sequencing (Materials and methods; Figure S1 in Additional file 1). Our full dataset therefore comprised 15 sites. Note that, for consistency, we refer to orthologous miRNAs according to how they are annotated in humans, for example, we use miR-376a-1 to denote both the human miRNA hsa-miR-376a-1 and the mouse miRNA mmu-miR-376a*. A list of species-specific miRNA identifiers, following the annotations provided by Meunier *et al*. [3], is included in Table S1 in Additional file 2.

### Deep conservation of site-specific A-to-I miRNA editing

Strikingly, our analysis revealed three site-specific miRNA editing events that were shared between mammals and birds (Table 1), thus implying that the resulting miRNA variants have persisted in the transcriptome for more than 320 million years [20]. One of these events, editing of miR-455 at position 17, had been verified by ADARB1 overexpression experiments in human cell lines [13]. Editing of miR-140* and miR-301a had not been reported previously, consistent with our detection of a strong editing signal in opossum and chicken, but not in human and mouse (Table 1). Nevertheless, there is evidence that ADARs bind these sequences also in placental mammals, as both miRNAs are upregulated in *Adarb1*^-*/*-^ or *Adar*^-*/*-^, *Adarb1*^-*/*-^ knockout mouse embryos [17], suggesting that they might be subject to editing-independent regulation by ADARs [21]. Potentially, editing of these miRNAs could therefore be a side effect of other regulatory functions performed by ADARs, which might in turn explain why the deeply conserved editing sites of miR-140*, miR-301a and miR-455 all lie outside of the seed sequence and other parts of the miRNA that may influence targeting properties [22]. The conserved roles of ADARs as regulators of miRNA biogenesis and function therefore likely go beyond site-specific editing of bases involved in target recognition.

Most (73%) of the conserved edited sites identified in our study do, however, fall within the seed region (Table 1). As a consequence, editing is expected to redirect these affected miRNAs to new sets of target genes [11]. Several editing events within the seed were found to be much more ancient than previously recognized: editing of miR-27a was found in placental mammals and platypus, thus presumably dating back at least 220 million years, while editing of miR-187*, miR-497 and miR-1251 was shared between placental mammals and marsupials, whose last common ancestor lived 180 million years ago [20].

**Figure 1:**
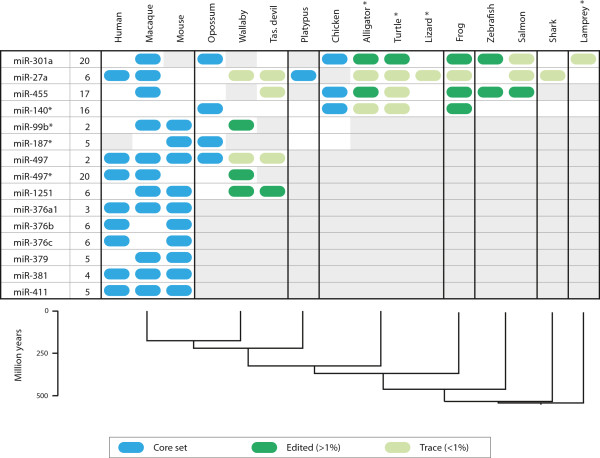
**Conservation of miRNA editing in vertebrates.** The identifier of the edited miRNA and the position at which editing occurs are indicated on the left. Observed miRNA editing with a frequency of >1% is indicated as blue boxes for the species in the core set (Table 1) and as dark green boxes for the 10 additional species. For completeness, cases where A-to-G mismatches were present at a frequency <1% (‘trace reads’) are shown as light green boxes, although it should be noted that such trace events cannot be readily distinguished from sequencing errors. Gray shading indicates that the presence of miRNA editing could not be assessed due to a lack of sequencing reads for the miRNA in question (which might be explained by the rigorous filtering criteria, lack of expression in the investigated samples or absence of the miRNA in the genome). An asterisk following the species name indicates that no quality scores were available for the species in question. Divergence times were taken from the TimeTree database [20]. Note that the analysis presented here was performed using a stringent detection algorithm to avoid false positives; editing of a given miRNA might therefore have escaped detection in some species (compare Figure 3).

Furthermore, we noticed that some miRNAs with conserved edited sites had annotated orthologs also in more distantly related species [2]. To further refine the dating of our identified editing events, we analyzed published small RNA sequencing data (Materials and methods) from 10 additional vertebrate species, including representatives of marsupials, reptiles, frogs, bony fishes, sharks and lampreys (Figure 1). Remarkably, we found that miR-301a and miR-455 were edited in bony fishes, implying that both miRNAs have experienced site-specific RNA editing during the past 450 million years [20]. Our results hint at an even earlier emergence of miR-27a and miR-301a editing, which might also be shared with sharks and lampreys, respectively, although the signal is too weak for us to exclude completely that it stems from sequencing errors. Nevertheless, given that we observed A-to-G mismatches at the same site in multiple species, it appears probable that these mismatches do represent genuine cases of miRNA editing. The deep conservation of site-specific miRNA editing strongly suggests that ADARs are important regulators of miRNA biogenesis and that, in some cases, their binding sites have been maintained by purifying selection throughout vertebrate evolution.

### Expansion of edited miRNAs in placental mammals

Not all miRNA editing events are ancient: we found six cases of miRNA editing (miR-376a-1, miR-376b, miR-376c, miR-379, miR-381 and miR-411) that were limited to placental mammals and that therefore represent evolutionary novelties (Figure 1). Interestingly, all six miRNAs were grouped into the same family based on sequence similarity [3] and are located within a single miRNA cluster [23]. Some of these miRNAs are transcribed together as part of a polycistronic primary sequence [11]. Because none of the six miRNAs has annotated orthologs in marsupials or other more distantly related species [2,3], the family most likely appeared and expanded recently, thereby substantially adding to the repertoire of edited miRNAs in humans and other placental mammals. Similar expansions might have taken place in other lineages, but we currently have limited power to detect them.

In the six placental-specific miRNAs, the observed editing sites are not at corresponding positions (Figure S2 in Additional file 1), which is likely explained by the presence of multiple editing sites within the miRNA precursor that are visible or invisible in the mature miRNA depending on the arm from which it is derived [11]. All observed editing events do, however, occur within the seed sequence and therefore have the ability to redirect the miRNA to a new set of targets. For one miRNA in particular, miR-376a-1, it has previously been demonstrated that the edited and unedited forms silence distinct sets of target genes [11]. The recent expansion of this edited miRNA family might therefore have had functional consequences for target gene expression and thus potentially contributed to lineage-specific characteristics of placental mammals.

### Combined effects of miRNA editing and alternative 5′ cleavage on miR-411 target specificity

**Figure 2:**
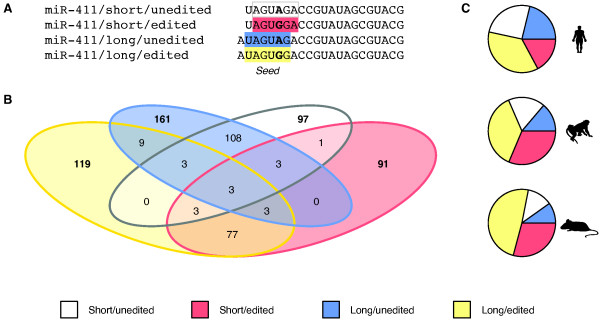
**Conserved editing and 5′ length variation of miR-411. (A)** As a result of A-to-I editing (here represented as A-to-G) and alternative 5′ cleavage, the miR-411 precursor gives rise to four variants with distinct seed sequences. The short, unedited variant is the annotated form. **(B)** Venn diagram of the predicted targets for each miR-411 variant. Targets were predicted with TargetScan release 6.0 [25] and were required to be present in at least 10 species, including human, macaque and mouse. **(C)** Relative abundance of the four miR-411 variants in (from top to bottom) human, macaque and mouse, when considering reads from all five tissues together.

In addition to editing of miRNA seed sequences, miRNA variants with novel targeting properties might also arise as a result of alternative 5′ cleavage when the mature miRNA is excised from its precursor [18,24]. One miRNA in our dataset, miR-411, exhibited both substantial miRNA editing and 5′ length variation, resulting in four miRNA variants with distinct seed sequences (Figure 2A) and, as a consequence, largely non-overlapping sets of predicted target genes (Figure 2B). The miR-411 variants were expressed in roughly similar proportions (Figure 2C) and at relatively high levels; miR-411 fell within the 100 most highly expressed miRNAs in all investigated tissues, except mouse kidney [3].

Moreover, we observed a conserved association between the choice of 5′ cleavage site and editing: among unedited human transcripts, we found that 54% started at the same position as the annotated miRNA [2,3], while 45% started at position -1 (Figure 2C). Among the edited transcripts, on the other hand, the proportions were 32% and 68% (*P* < 10^-15^, χ^2^-test, excluding reads with other start sites), showing that the location of the 5′ cleavage site and the presence of editing are not independent of each other in our data. The same skew was present for macaque and mouse (*P* < 10^-15^ in both cases). In principle, this observation might be explained by biases during library preparation, which might lead to preferential amplification of some miR-411 variants over others [26]. However, Chiang *et al*. noted a similar association between miR-411 editing and 5′ variation in independently generated mouse data [18], which makes this explanation unlikely. Instead, the observed skew suggests that the base change introduced by the editing machinery influences subsequent processing steps of the miR-411 precursor, presumably by altering structural motifs within the hairpin [27]. The effects of A-to-I editing on the miRNA pathway therefore range from a dramatic reduction of processing efficiency resulting in loss of miRNA expression [9], to a more subtle influence on the choice of cleavage site, which nevertheless alters the mature miRNA sequence and therefore can have a profound and evolutionarily conserved impact on miRNA target specificity.

### Tissue-specific effects on miRNA editing frequencies

**Figure 3:**
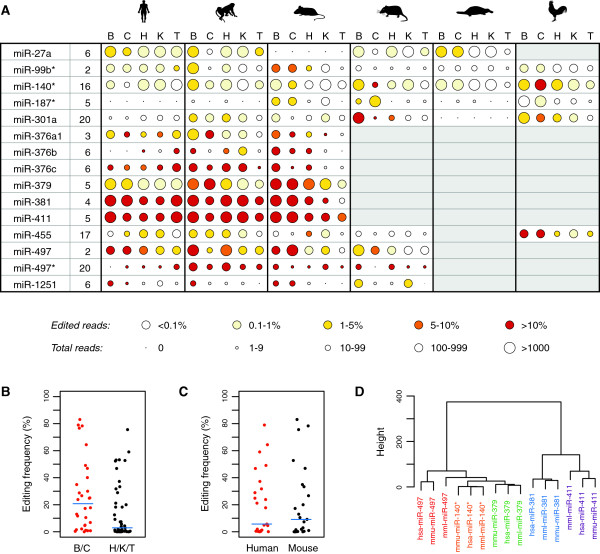
**Frequency of miRNA editing across tissues and species. (A)** Estimated miRNA frequencies in brain (B), cerebellum (C), heart (H), kidney (K) and testis (T). From left to right, the animal silhouettes represent human, macaque, mouse, opossum, platypus and chicken. The miRNA identifiers are given in the left-hand column and are followed by the position of the edited site within the mature or star miRNA. The color of each circle corresponds to the proportion of edited reads, while the size of the circle corresponds to the total number of reads for the miRNA in question. Note that these data are not normalized and that expression levels therefore should not be compared across samples. Gray shading indicates the absence of an annotated miRNA ortholog in that particular species, based on the information in Table S1 in Additional file 2. The data used to generate this figure are provided in Table S2 in Additional file 2. **(B)** Comparison of editing frequencies in neural (brain and cerebellum) versus non-neural (heart, kidney and testis) tissues in human, macaque and mouse, for miRNAs with at least 10 reads in all relevant samples. The median frequency is indicated by a blue line. **(C)** Comparison of editing frequencies in human and mouse samples for the same miRNAs as in (B). The median frequency is indicated by a blue line. **(D)** Hierarchical clustering of the same miRNAs as in (B), based on editing frequencies in five tissues in human (hsa), macaque (mml) and mouse (mmu). Orthologous miRNAs have been given the same color. The clustering was performed using the R function hclust and Ward’s method.

It has been suggested that miRNA editing is more common in the brain [13,14], although other studies found similar editing frequencies in neural and non-neural tissues [8,10]. To evaluate miRNA editing patterns across tissues and species, we estimated miRNA editing frequencies for each of the six species and five tissues (cortex or whole brain without cerebellum, hereafter referred to as brain, cerebellum, heart, kidney and testis) in our core dataset [3]. Rather than relying on the detection pipeline, we based our estimates on reads that aligned perfectly to either the edited or the unedited sequences of the miRNAs identified above (Materials and methods). The reason for using this approach, rather than taking the read counts given by the detection pipeline, was that the latter introduced biases by treating edited and unedited reads differently; for example, the pipeline would discard edited reads with one additional mismatch, whereas unedited reads with a single mismatch would be retained. In addition, our remapping method proved to be more sensitive and was able to detect editing in additional species, for example, of miR-379 in human, and of miR-497* in mouse and opossum (Figure 3; Table S2 in Additional file 2). Although the method could be liable to cross-mapping [28], this was not an issue for our set of miRNAs, since only 1,927 out of 713,195 remapped reads (0.3%) had an additional perfect match elsewhere in the genome.

The most highly edited miRNA in our dataset was miR-411, for which editing reached 83% (2,225 edited and 454 unedited reads) in mouse cerebellum (Table S2 in Additional file 2). This finding already hinted that miRNAs might indeed reach higher levels in neural tissues, but to test this more formally, we considered all miRNAs with at least 10 reads in each tissue (miR-140*, miR-379, miR-381, miR-411 and miR-497). For these miRNAs, we did indeed observe higher levels of miRNA editing in brain and cerebellum compared to the other tissues (*P* = 0.012, Mann-Whitney test; Figure 3B). Importantly, however, we also observed high editing frequencies elsewhere, for example, in human kidney where miR-411 was edited at 59% (559 edited and 387 unedited reads) and miR-381 at 32% (199 edited and 428 unedited reads). While our results confirm the general tendency for increased miRNA editing in neural tissues [13,14], consistent with what has been observed for mRNAs [29,30], they therefore also highlight the necessity of studying several tissues in order to fully understand how miRNA editing modulates mammalian gene regulation. It will also be of interest to study how editing varies across cell types to determine whether editing levels are uniform or whether low overall frequencies of editing might correspond to extensive editing restricted to a limited number of cells.

We did not find a significant difference between editing levels in humans and mice (*P* = 0.79, Wilcoxon signed rank test; Figure 3C), contrary to an earlier report [31], but consistent with more recent results [13]. To further investigate whether there are species-specific effects on editing patterns, we performed hierarchical clustering, which separated the data according to miRNA identity, rather than species (Figure 3D), thus indicating that broad patterns of miRNA editing are stable across species.

### Conserved increase of miRNA editing with age in humans and macaques

Editing frequencies are not only tissue-dependent, but also appear to be developmentally regulated, since the degree of miRNA editing tends to increase after birth and during early postnatal development in mouse [14,32]. We wished to investigate whether this trend was present for our set of conserved miRNA editing events and, if so, whether the observed patterns were consistent across species. To this end, we analyzed data from human and macaque brain, which covered 12 time points of postnatal development and aging in both species [33].

**Figure 4:**
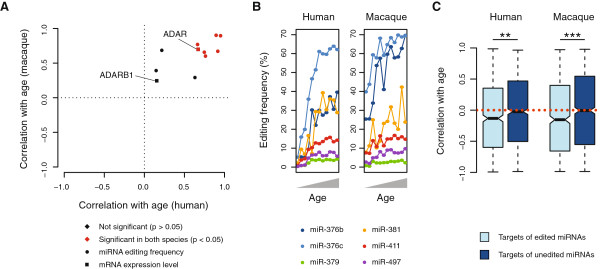
**Changes in miRNA editing frequencies during primate postnatal development and aging. (A)** Spearman correlation coefficients for miRNA editing frequencies and age in human and macaque (see main text for details). The respective correlations for ADAR and ADARB1 mRNA expression levels are indicated as squares. Cases where the correlation coefficients were significant in both species are highlighted in red. **(B)** Editing frequencies for the six significant miRNAs (see (A)) in samples from different ages, with younger individuals on the left and older on the right. **(C)** Distribution of correlation coefficients for age and expression levels, calculated for mRNAs predicted to be targeted either by edited or unedited miRNAs. If expression is independent of age, the expected correlation coefficient is 0, as indicated by the dotted line. Statistical significance is indicated by double (0.001 < *P* < 0.01) or triple (*P* < 0.001) asterisks.

Out of the nine edited miRNAs for which we had sufficient read coverage, seven showed a significant positive correlation between editing frequency and age in humans, and seven in macaques (Figure 4A; *P* < 0.05, Spearman correlation with Benjamini-Hochberg correction for multiple testing). Editing of miR-376b, miR-376c, miR-379, miR-381, miR-411 and miR-497 was significantly correlated with age in both species, demonstrating that the age-related increase of editing frequencies at specific sites is conserved between species (Figure 4B). This idea is further supported by the overlap between our results and those of Ekdahl *et al*. [14], who studied miRNA editing in the developing mouse brain: all five miRNAs from our dataset that were included in the mouse study were more highly edited in postnatal compared to embryonic mouse samples [14], and also showed a significant age-related increase of editing frequencies in at least one species in our analysis. The trend was not explained by a general increase in transcription errors or other mismatches in older individuals, since we did not find elevated A-to-G mismatch frequencies at nearby positions within the affected miRNAs (Figure S3 in Additional file 1).

We hypothesized that the increase in miRNA editing might be explained by higher abundance of ADARs in older individuals and therefore calculated the correlations for ADAR and ADARB1 mRNA levels with age, based on data from the same individuals that were used for the miRNA analysis [33]. We found that expression of ADAR, but not ADARB1, was significantly correlated with age in both humans and macaques (Figure 4A), suggesting that ADAR might be responsible for the increase in miRNA editing. Consistent with this, miR-376b, miR-381 and miR-411 are thought to be edited primarily by ADAR [12,13]. However, the editing sites in miR-379 and miR-497 appear to be targets of ADARB1 [12,13]. Furthermore, a recent study of mRNA editing in primates found a similar increase in editing frequencies with age, but without observing a consistent increase in ADAR expression [34]. As a consequence, changes in ADAR expression might go some way toward explaining why miRNA editing frequencies are higher in older individuals, but additional regulatory mechanisms are likely to be involved.

### Reduced expression of genes targeted by edited miRNAs

When editing occurs within the seed sequence, it is expected to influence miRNA targeting. Such an effect has been demonstrated for miR-376a-1 [11,14], but other miRNAs are less well studied in this regard. The developmental dataset from Somel *et al*. [33] provided us with an opportunity to study the regulatory implications of miRNA editing, since it included miRNA and mRNA expression data from the same individuals. We therefore predicted target genes using TargetScan [25] for the edited and unedited forms of the six miRNAs for which we had detected an age-related increase in editing frequency. To enrich for functional interactions, we only included genes with a detected target site in at least 10 species, including human and macaque.

If miRNA editing contributes to gene regulation, those genes that are targeted by edited miRNAs should show decreased expression as editing frequencies increase. Genes targeted by the unedited forms of these miRNAs, on the other hand, would be expected to show increased expression as the unedited miRNAs become less abundant, although the relative difference in abundance might be too minor to influence target gene expression. These predictions corresponded well with our observations (Figure 4C): genes that were predicted to be targets of edited miRNAs were significantly more likely to decrease in expression with age, compared to genes targeted by the unedited form of the same miRNAs, in both humans and macaques (*P* = 0.001 and *P* = 0.0003, Mann-Whitney test). Thus, miRNA editing likely contributes to age-related gene expression changes in the primate brain.

### Downregulation of miRNA editing in human cancers

Possibly, the high levels of editing in neural tissues and older individuals, compared to the low levels in testis and younger individuals, could reflect a general association between A-to-I editing and the rate of cell proliferation. To investigate this further, we decided to extend the characterization of our set of conserved miRNA editing events to include differences between normal and cancerous tissue samples. Editing of long RNAs is known to be altered in human cancers, with general hypoediting of transposable elements and gene-specific increases or decreases of mRNA editing [35]. While the general patterns of miRNA editing in cancer remain unknown, decreased editing of miR-376a-1 has been established as a contributing factor in the formation of human gliomas [15].

**Figure 5:**
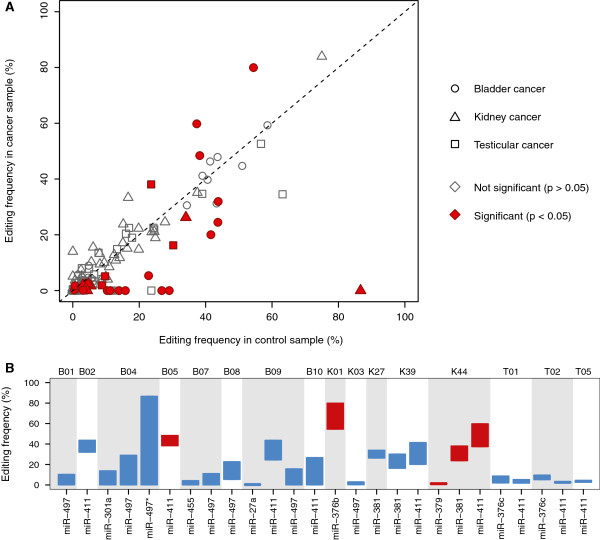
**Comparison of editing frequencies in samples from cancerous and healthy tissues. (A)** Each point represents the estimated editing frequency of a single miRNA in a matched sample (cancer and control) from the same patient. Cases where a significant difference in editing was detected between the two conditions (χ^2^-test with Benjamini-Hochberg correction) are highlighted in red. **(B)** Summary of the significant cases in (A). Sample identifiers consist of a letter giving the cancer type and a number that refers to the patient identifier in the original study. Blue bars represent significant downregulation of miRNA editing in cancer samples, with the top of the bar corresponding to the editing frequency in the control sample and the bottom of the bar corresponding to the editing frequency in the cancer sample. Red bars represent significant editing upregulation, with the bottom of the bar corresponding to the editing frequency in the control sample and the top of the bar corresponding to the editing frequency in the cancer sample.

We focused our analysis on matched samples (healthy and cancerous) from 10 patients with bladder cancer, 10 with kidney cancer and 7 with testicular cancer [36,37]. Overall, our results suggest that downregulation of miRNA editing is a common phenomenon in human cancers, consistent with our hypothesis that low editing frequencies are associated with fast cell proliferation (Figure 5A): of the 213 miRNA-patient combinations for which we had sufficient read coverage, the estimated editing frequency was lower in the cancer sample compared to the control sample from the same individual in 136 cases (64%, *P* = 6.4 × 10^-5^, binomial test). The trend was even stronger when we only considered instances where the difference for a particular miRNA and sample pair was significant (*P* < 0.05, χ^2^ test with Benjamini-Hochberg correction for multiple testing): using this criterion, we observed 21 cases of downregulation and 5 cases of upregulation (81%, *P* = 0.0025, binomial test). The dataset was unfortunately too limited to determine whether there are reproducible differences in editing patterns between different types of tumors and to what extent the behavior of individual miRNAs might be linked to interferon regulation, which affects ADAR but not ADARB1 [38].

Within a single individual, changes in editing frequencies were always in the same direction. For example, we found a significant downregulation of miR-27a, miR-411 and miR-497 in bladder cancer patient B09 and a significant upregulation of miR-379, miR-381 and miR-411 in kidney cancer patient K44 (Figure 5B). If the trend holds also for larger sample sizes, it would be of great interest to evaluate whether global upregulation or downregulation of miRNA editing is associated with different clinical outcomes for these cancer types, given that higher-grade brain tumors were shown to be associated with stronger reduction of miR-376a-1 editing [15]. Considering the potentially substantial downstream effects of altered miRNA regulation, it appears probable that additional edited miRNAs beside miR-376a-1 could contribute to transcriptomic and phenotypic characteristics of human tumors.

## Discussion

In recent years, it has become clear that ADAR enzymes edit specific nucleotides within mammalian miRNAs and that the resulting sequence alterations can influence the expression of the miRNAs themselves, as well as the expression of the mRNAs they target. However, the existence of miRNA editing, or even its demonstrated regulatory potential, does not automatically imply that this process confers an evolutionary advantage to the organism. Considering that ADARs use dsRNA molecules as their substrate and that the formation of a dsRNA structure is an important step of miRNA biogenesis, editing of primary miRNA transcripts might sometimes occur by chance, without serving any regulatory purpose. The likelihood of accidental editing might be further increased by interactions between ADAR and Dicer, a central component of the miRNA biogenesis pathway, which bring the editing enzyme into the proximity of its potential substrates [39]. Indeed, edited miRNAs are not unique to mammals, but have also been observed in other species such as *Drosophila melanogaster* and *Caenorhabditis elegans*[40,41]. The wide phylogenetic distribution suggests that miRNA editing might be a common consequence of the coexistence of miRNAs and ADARs, although it does not in itself offer any clues regarding the regulatory importance of miRNA editing.

One approach to distinguish between off-target effects and biologically meaningful miRNA editing is to consider the evolutionary history of individual editing events, on the assumption that only functional editing will be maintained by purifying selection. In this study, we have identified 15 conserved miRNA editing events; all of these have been conserved for a minimum of 90 million years and two of them are shared between mammals, birds, reptiles and bony fishes, whose last common ancestor lived 450 million years ago [20]. For these miRNAs, the edited variants clearly are not created at random, but represent evolutionarily stable modifications of the transcriptome. Interestingly, our number of conserved editing events is comparable to estimates based on single species: although differences in methodology, choice of samples, data quality and read coverage can complicate the direct comparison of results from different studies, we nevertheless note that recent studies of miRNA editing in human and mouse, which did not rely on evolutionary conservation as a detection criterion, reported between 8 and 24 events per species [12-14,17,18]. The tendency for edited miRNAs to be shared across species stands in stark contrast to the evolution of A-to-I editing in general: in a recent study, Pinto *et al*. [42] investigated the evolutionary conservation of over 1.4 million known human editing events, of which 52,000 occurred outside of transposable elements [43-45], using long RNA sequencing data from several mammalian species, and were able to identify a total of only 59 conserved sites. Although miRNA editing might account for but a minuscule fraction of the total A-to-I editing activity in a single species, the exceptional conservation of these events nevertheless places miRNA editing as one of the main mechanisms through which ADAR enzymes have contributed to the generation of alternative transcripts during mammalian evolution.

Conceivably, the ability to be edited might be retained for reasons not related to editing itself, such as if the miRNA requires a specific sequence or secondary structure, which coincidentally happens to contain motifs that are recognized by ADARs. However, this is an unlikely explanation for several reasons. Firstly, as demonstrated here and elsewhere, miRNA editing can dramatically alter target specificity [11,13-15], as well as miRNA expression levels [9,16]. That such transcriptomic changes would be neutral is highly improbable, especially in the light of the strong constraints on miRNA evolution [3]. Secondly, unlike protein sequences, miRNA sequences are not meaningful in themselves, but only in relation to motifs within the mRNAs they regulate. The functions of editable miRNAs could therefore be equally well carried out by miRNAs that cannot be edited. Thirdly, editing activity could easily be abolished, either through a single substitution of the edited adenosine into any other nucleotide, or by other mutations that turn the primary miRNA transcript into an unsuitable editing substrate, similar to the majority of mammalian miRNAs. Taken together, it is therefore difficult to imagine a situation where a particular miRNA sequence would be so advantageous, or where the removal of editing motifs would be so complicated, that miRNA editing would be maintained in spite of its deleterious effects. As a consequence, the conservation of miRNA editing is most likely due to its incorporation into the regulatory networks of the cell.

Our study further shows that miRNA editing at conserved positions is highly variable with regards to tissue, age and disease state. Importantly, this variation is not random and we observe a significant enrichment of edited reads in neural tissues, older individuals and healthy relative to cancerous samples. Moreover, we show that tissue-specific and age-specific patterns of miRNA editing are shared across species and that the reduction of miRNA editing in human cancer is consistent across several types of tumors. The dynamic, yet evolutionarily stable, nature of A-to-I editing activity, in combination with the ability of edited miRNAs to simultaneously modulate the expression of multiple target genes and the possibility of fine-tuning this response through the evolution of miRNA motifs within each target, thus all likely contribute to make ADAR-mediated miRNA editing a powerful and versatile mechanism for the precise control of gene expression in mammals and other vertebrates.

## Conclusions

We present the first detailed survey of the evolution of miRNA editing, based on data from several mammalian and non-mammalian species. Our results show that while miRNA editing is considerably less frequent than editing of other RNAs within a single species, the strong conservation of many miRNA editing events means that edited miRNAs are highly overrepresented in the total conserved editome. Moreover, we find that conserved age-related changes in miRNA editing frequencies contribute to the modulation of gene expression during primate brain development, thus illustrating the functional implications of miRNA editing on downstream gene regulation. Together, our findings underline the importance of site-specific miRNA editing as a mechanism to generate functional miRNA variants in mammalian evolution.

## Materials and methods

### Identification of edited miRNAs

We based our analyses on small RNA sequencing data from Meunier *et al*. [3]. After removing the adapter sequences, we filtered the reads to only retain those with a length of 15 to 28 nucleotides and a minimum quality score of 30 (corresponding to an error rate of 0.1%) at each position. We mapped these reads to genomic sequences from Ensembl release 68 [46] using Bowtie [47], allowing one mismatch and only keeping reads with a single best alignment (-v 1 -a -m 1 --best -strata). Previous studies found that editing of multiple positions within a single mature miRNA was very rare [18] and that the inclusion of reads with two mismatches did not increase the power to detect editing events [13]. The mapping was repeated for reads where either one or two bases had been removed from the 3′ end to allow for 3′ modifications [18,48].

To call putative miRNA editing events, we first identified mismatches that mapped within and on the same strand as annotated mature or star miRNA sequences [3]. To exclude 5′ and 3′ modifications, we removed mismatches that occurred within the first one or last two bases of the read or the annotated sequence. We further discarded sites that were not covered by at least one perfectly mapping read or for which we detected more than one mismatch type at a frequency above the sequencing error rate (0.1%). For each candidate site, we required the matching and mismatching variant to be represented by at least five reads each, corresponding to at least 5% of the total read count for that miRNA in a single tissue.

The miRNAs were grouped into families based on the annotations provided by Meunier *et al*. [3] and aligned using MUSCLE [49]. For putatively edited miRNAs with multiple orthologs in a single species, only the most similar ortholog was retained, that is, the sequence with the fewest mismatches within the mature miRNA. Based on the alignments, we filtered out candidate editing events that occurred at non-conserved sites, since the relaxed constraint at these sites might make them more likely to harbor SNPs. For the remaining events, we required that they were found in at least two species, although not necessarily in the same tissue.

### Overlap with polymorphic sites

Our set of putative editing events was compared to SNP data from human and opossum to determine whether some candidates might be explained by polymorphisms. As our human dataset, we used common SNPs from dbSNP build 137 [19]. For opossum, we prepared genomic DNA, by chloroform extraction, from the same opossum individual for which we had previously generated the brain small RNA library [3]. The genomic regions corresponding to the miRNAs found to be edited in this sample were verified by Sanger sequencing (Figure S1 in Additional file 1).

### Detection of miRNA editing in additional species

We sequenced small RNAs from adult brain and heart of the Western clawed frog (*Xenopus tropicalis*). RNA was extracted from 20 to 30 mg of tissue, using the miRNeasy mini kit (Qiagen, Hilden, Germany) according to the manufacturer’s instructions. The RNA quality was checked on a Fragment Analyzer (Advanced Analytical Technologies, Ames, Iowa, USA). We used 2 μg of total RNA to prepare the small RNA libraries. Following purification of small RNA on a 15% TBU acrylamide gel, we generated the libraries using the Illumina TruSeq Small RNA Sample Prep Kit and assessed the quality on a Fragment Analyzer. The libraries were sequenced on an Illumina HiSeq 2500 instrument, to yield approximately NN single-end reads of 101 nucleotides per library.

In addition, we analyzed published data from tammar wallaby (*Macropus eugenii*), Tasmanian devil (*Sarcophilus harrisii*), American alligator (*Alligator mississippiensis*), painted turtle (*Chrysemys picta bellii*), anole lizard (*Anolis carolinensis*), zebrafish (*Danio rerio*), Atlantic salmon (*Salmo salar*), whitespotted bamboo shark (*Chiloscyllium plagiosum*) and sea lamprey (*Petromyzon marinus*) [50-56]. Only samples from healthy, wild-type individuals were considered. If quality scores were available, we required a minimum score of 20 at each position of the read. In addition, we required that the sites that were evaluated in terms of editing had a minimum score of 30. RNA sequencing reads were mapped onto the relevant genome, while allowing one mismatch [46,57-60]. Because some genomes were incompletely assembled, we allowed reads to map to up to five locations, but only kept reads from the best stratum. A site was considered edited if the matching and mismatching variant were covered by at least one read each, corresponding to at least 1% of the total reads covering the site in that sample. Orthologous cases of miRNA editing were determined by requiring that the corresponding human annotated mature or star sequence mapped to the same location with no more than two mismatches. A more detailed account of the samples and genome assemblies used in this analysis is included in Table S3 in Additional file 2.

### Estimation of tissue-specific editing frequencies

To refine our estimates of tissue-specific miRNA editing, we extracted the genomic sequences of all miRNAs with conserved editing, along with 10 nucleotides on either side of the annotated mature or star sequence. We then remapped the quality-filtered reads from the six species and five tissues in our core dataset [3] against the edited and unedited form of each miRNA. Only reads that mapped without mismatches and spanned the edited site were included in downstream analyses. To test for potential cross-mapping, we remapped these reads against the relevant genome (bowtie -v 0) and calculated the number of reads with additional perfect matches outside our set of 15 miRNAs.

### miRNA editing and target gene expression in primate time course data

We estimated editing frequencies based on small RNA sequencing data from Somel *et al*. [33], using the same method as for the tissue specificity analysis described above. As a control, we also aligned the reads against a third sequence with ‘fake editing’, where we had replaced the closest A to the genuine editing site with a G. For those samples where replicates were available, all reads were analyzed jointly. To avoid artifacts caused by insufficient read coverage, we only considered miRNAs that were detected in all samples and for which we could identify a minimum of 10 edited reads per species. We calculated the Spearman correlation coefficient for miRNA editing frequency and age of the individual for each miRNA and corrected the *P* values for multiple testing using the Benjamini-Hochberg method. The same analysis was also performed for normalized ADAR and ADARB1 mRNA expression [33].

We predicted miRNA target sites using TargetScan release 6.0 [25], using default settings and the provided UTR alignments. To enrich for authentic interactions, we focused our analysis on genes for which a given target site was detected in at least 10 species, including human and macaque. We excluded genes that were predicted targets of both edited and unedited miRNAs. For the predicted targets, we then calculated Spearman correlation coefficients as detailed above, based on normalized mRNA expression data from Somel *et al*. [33].

### Analysis of human cancer samples

Small RNA sequencing data from cancer patients were taken from Zhou *et al*. [36] and Li *et al*. [37], and analyzed using the same method as for the tissue specificity analysis. To ensure sufficient read depth to detect differential miRNA editing, we required each miRNA to be represented by at least 100 reads in a given individual.

### Data availability

The *Xenopus* small RNA sequencing data have been submitted to the NCBI Gene Expression Omnibus with accession number GSE56680.

## Abbreviations

ADAR: adenosine deaminase acting on RNA; dsRNA: double-stranded RNA; miRNA: microRNA; SNP: single-nucleotide polymorphism; UTR: untranslated region.

## Competing interests

The authors declare that they have no competing interests.

## Authors’ contributions

MW conceived of the study, performed all analyses and wrote the manuscript with input from all authors. AL carried out the sequencing of opossum DNA. JH and DV prepared *Xenopus* small RNA libraries. HK supervised the project. All authors read and approved the final manuscript.

## Supplementary Material

Additional file 1 **Figure S1.** Verification of the opossum genomic sequence by Sanger sequencing. **Figure S2.** Editing sites in a group of related miRNAs. **Figure S3.** Mismatch frequencies during primate development at edited and control sites in six miRNAs. Click here for file

Additional file 2 **Table S1.** Species-specific miRNA identifiers based on the annotations by Meunier *et al*. [3]. **Table S2.** Frequencies of miRNA editing in five tissues and six species. **Table S3.** Overview of RNA sequencing datasets and genome assemblies that were used, together with the core dataset [3], to generate Figure 1. Click here for file
