# Supplementary material for: Conserved microRNA editing in mammalian evolution, development and disease
Source: Genome Biol. 2014 Jun 25;15(6):R83. doi: 10.1186/gb-2014-15-6-r83 (PMC4197820; doi:10.1186/gb-2014-15-6-r83)
Supplement: Additional file 1 — Figure S1. Verification of the opossum genomic sequence by Sanger sequencing. Figure S2. Editing sites in a group of related miRNAs. Figure S3. Mismatch frequencies during primate development at edited and control sites in six miRNAs. [file gb-2014-15-6-r83-S1.docx]

**Supplementary Figure 1.** Verification of the opossum genomic sequence by Sanger sequencing. DNA was extracted from the same opossum individual for which the brain small RNA library had previously been generated (Meunier et al, 2013). For each opossum miRNA, the forward and reverse primers are given, along with relevant sections of the output from the forward (left) and reverse (right) sequencing reactions.

**Supplementary Figure 2.** Editing sites in a group of related miRNAs. Stem-loop structures were taken from miRbase release 20 (Kozomara et al, 2011). The mature sequence is highlighted in red following the annotations from Meunier et al (2013) and the edited site is indicated by an arrow.

**Supplementary Figure 3.** Mismatch frequencies during primate development at edited and control sites in six miRNAs. **A.** A-to-G mismatches in human data. Mismatch frequencies at the genuine editing site within each miRNA are shown in the left section of the graph (these data are also displayed in Fig. 4B). In the right section are the results from an analogous analysis performed for the nearest adenosine within the same miRNA. **B.** Same as in A, but for the macaque dataset.
